# Comparing Medical Record Abstraction (MRA) Error Rates in an Observational Study to Pooled Rates Identified in the Data Quality Literature

**DOI:** 10.21203/rs.3.rs-2692906/v1

**Published:** 2023-03-27

**Authors:** Maryam Y. Garza, Tremaine B. Williams, Songthip Ounpraseuth, Zhuopei Hu, Jeannette Lee, Jessica Snowden, Anita C. Walden, Alan E. Simon, Lori A. Devlin, Leslie W. Young, Meredith N. Zozus

**Affiliations:** University of Arkansas for Medical Sciences; University of Arkansas for Medical Sciences; University of Arkansas for Medical Sciences; University of Arkansas for Medical Sciences; University of Arkansas for Medical Sciences; University of Arkansas for Medical Sciences; University of Colorado Denver, Anschutz Medical Campus; Centers for Disease Control and Prevention; University of Louisville; University of Vermont; University of Texas Health Science Center at San Antonio, Joe R. & Teresa Lozano Long School of Medicine

**Keywords:** medical record abstraction, data quality, clinical research, clinical data management, data collection

## Abstract

**Background:**

Medical record abstraction (MRA) is a commonly used method for data collection in clinical research, but is prone to error, and the influence of quality control (QC) measures is seldom and inconsistently assessed during the course of a study. We employed a novel, standardized MRA-QC framework as part of an ongoing observational study in an effort to control MRA error rates. In order to assess the effectiveness of our framework, we compared our error rates against traditional MRA studies that had not reported using formalized MRA-QC methods. Thus, the objective of this study was to compare the MRA error rates derived from the literature with the error rates found in a study using MRA as the sole method of data collection that employed an MRA-QC framework.

**Methods:**

Using a moderator meta-analysis employed with Q-test, the MRA error rates from the meta-analysis of the literature were compared with the error rate from a recent study that implemented formalized MRA training and continuous QC processes.

**Results:**

The MRA process for data acquisition in clinical research was associated with both high and highly variable error rates (70 – 2,784 errors per 10,000 fields). Error rates for the study using our MRA-QC framework were between 1.04% (optimistic, all-field rate) and 2.57% (conservative, populated-field rate) (or 104 – 257 errors per 10,000 fields), 4.00 – 5.53 percentage points less than the observed rate from the literature (p<0.0001).

**Conclusions:**

Review of the literature indicated that the accuracy associated with MRA varied widely across studies. However, our results demonstrate that, with appropriate training and continuous QC, MRA error rates can be significantly controlled during the course of a clinical research study.

## Background

Computers have been used in clinical studies since the early 1960s, although initial attempts to integrate them into research work flows were experimental and sporadic.^1,2^ The early application of computers to health-related research spawned a plethora of methods for collecting and preparing data for analysis.^3,4^ These activities continue to be evaluated by metrics of cost, time, and quality.^5^ While cost and time affect the feasibility of research, and timeliness is certainly critical to the conduct and oversight of research, the scientific validity of research conclusions depends on data accuracy.^6^

Data accuracy is attributable to how data are collected, entered, and cleaned, or otherwise processed; and the assessment and quantification of data accuracy are crucial to scientific inquiry. Several texts describe approaches to general data quality management, independent of the domain area in which they are applied.^7–10^ These works focus on general methods for assessing and documenting data quality, as well as methods for storing data in ways that maintain or improve their quality, but do not provide sufficient details on data collection and processing methods applicable to specific industries and types of data.

Thus, they provide little to no guidance to investigators and research teams planning a clinical research endeavor or attempting to operationalize data collection and management.

In previous work, we extensively reviewed the clinical research data quality literature and identified gaps that necessitated a formal review and secondary analysis of this literature to characterize the data quality resulting from different data processing methods.^11^ Through this effort, we quantified the average, overall error rates attributable to 4 major data processing methods used in clinical research (medical record abstraction [MRA]), optical scanning, single-data entry, and double-data entry) based on the data provided within 93 peer reviewed manuscripts.^11^ Our results indicated that data quality varied widely by data processing method. Specifically, MRA was associated with error rates an order of magnitude greater than those associated with other data processing techniques (70–2,784 versus 2–650 errors per 10,000 fields, respectively).^11^ MRA was the most ubiquitous data processing method over the time period of the review. Unfortunately, in a more recent review of studies using MRA, the foundational quality assurance activities identified as important in MRA processes were rarely reported with clinical studies: describing and stating the data source within the medical record 0 (0%); use of abstraction methods and tools 18 (50%); controlling the abstraction environment, such as to prevent interruption and distraction 0 (0%); and attention to abstraction human resources such as minimum qualifications and training 15 (42%).^12^ Only 3 (8.3%) of the articles reported measuring and controlling the MRA error rate.^12^

In an effort to mitigate the inherent risks associated with MRA, we developed and employed a theory-based, quality control (QC) framework to support MRA activities in clinical research.^13^ Our MRA-QC framework involves standardized MRA training prior to study implementation, as well as a continuous QC process carried out throughout the course of the study. We implemented and evaluated the MRA-QC framework within the context of the Advancing Clinical Trials in Neonatal Opioid Withdrawal Current Experience (ACT NOW CE) Study^14^ to measure the influence of formalized MRA training and continuous QC on data quality. We then compared our findings with MRA error rates from the literature to better understand the potential influence of this framework on data quality in clinical research studies.

## Methods

### Preliminary Work: Comprehensive Literature Review

As described in a separate manuscript, a systematic review of the literature was performed to “identify clinical research studies that evaluated the quality of data obtained from data processing methods typically used in clinical research.”^11^ Here, we refer to this as the comprehensive literature review. The 93 manuscripts identified through the comprehensive literature review were categorized by data processing method (e.g., MRA, optical scanning, single-data entry, and double-data entry), and only those specific to MRA were considered for this meta-analysis.

### Information Retrieval: Meta-Analysis of MRA Error Rates

**Figure 1:**
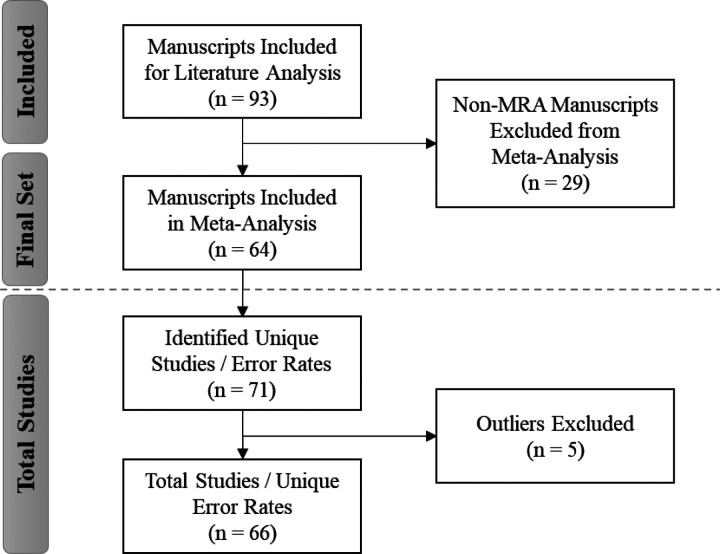
PRISMA Diagram: Identification of MRA-centric Literature for Meta-Analysis

Excluding manuscripts for which MRA was not the primary method of data collection from the comprehensive set yielded 64 MRA-centric manuscripts for inclusion in the meta-analysis (Fig. 1). For this evaluation, we referenced only this subset of MRA-centric studies to conduct a meta-analysis of the overall error rates as reported in the existing literature for comparison against the error rates derived for our study using the MRA-QC framework. Several manuscripts discussed multiple processing methods and/or studies, presenting unique error rates for each. Thus, within the set of 64 manuscripts, we identified 71 studies (or 71 unique error rates) for inclusion in the meta-analysis (see Additional File 1, Appendix A, Reference List A1 and Table A2).

Based on the residual and leave-one-out diagnostics,^15–17^ we identified 5 studies^18–22^ that were deemed to be potential outliers. Thus, these studies were removed for the final meta-analysis to obtain the estimate error rate for the literature reviews. In order to derive an overall MRA error rate for comparison with our study, we performed a meta-analysis of single proportions to derive an overall error rate from the literature based on an inverse variance method^23,24^ and generalized linear mixed model approach using the R package “*metafor*”.^25^ The general linear mixed model provides more robust estimates than traditional methods.^26^

### Comparison of MRA Error Rates to Results of Study Using MRA-QC Framework

Once the average MRA error rate across the literature was determined, we used that value to evaluate the effectiveness of a standardized MRA-QC framework implemented as part of a retrospective research study for which MRA was the sole method for data collection. Brie y, the MRA-QC framework was implemented within the context of the ACT NOW CE Study[a],^27^ a multicenter clinical research study sponsored by the National Institute of Health (NIH) through the Environmental Influences on Child Health Outcomes (ECHO) program.^28^ Thirty IDeA[b] States Pediatric Clinical Trials Network (ISPCTN)^29,30^ and NICHD[c] Neonatal Research Network (NRN)^31^ sites from across the U.S. participated in the study. Approximately 1,800 cases were abstracted across all study sites, of which a subset of cases (over 200) underwent a formalized QC process to identify data quality errors. Additional information on the ACT NOW CE Study,^14^ including details on the MRA training^32^ and QC process,^13^ has been published elsewhere.

The overall error rates for the ACT NOW CE Study were compared to error rates from the literature. The overall error rate for the ACT NOW CE Study^13^ was calculated using the same methodology used for calculating MRA error rates from the literature,^11^ based on the Society for Clinical Data Management’s (SCDM) Good Clinical Data Management Practices (GCDMP)^33^ Error Rate Calculation Framework (Formula 1).

### Number of Errors Detected


(1) $$\text{Error}\;\text{Rate}\;=\frac{\text{Number}\;of\;\text{Errors}\;\text{Detected}}{\text{Number}\;of\;\text{Fields}\;\text{Collected}}$$

We analyzed the data from each site within the ACT NOW CE Study separately to obtain site-specific error estimates and used meta-analysis to obtain a pooled error estimate.^34^ Next, we compared the all-field (optimistic) and populated-field (conservative) error rates from our study with the error estimate derived from the literature, based on a moderator analysis (subgroup analysis) using a Q-test to detect the difference between the two groups. The heterogeneity between studies were calculated and considered using the Higgins and Thompson’s I^2^ statistic.^35^

[a] ACT NOW CE Study: Advancing Clinical Trials in Neonatal Opioid Withdrawal Syndrome (ACT NOW) Current Experience: Infant Exposure and Treatment^27^

[b] IDeA: Institutional Development Awards Program. The IDeA program is a National Institutes of Health (NIH) program that aims to broaden the geographic distribution of NIH funding to support states that have historically been underfunded by providing resources to further expand research capacity across IDeA-eligible states^29,30^

[c] NICHD: The Eunice Kennedy Shriver National Institute of Child Health and Human Development is part of the NIH that supports (funds) the efforts of the Neonatal Research Network (NRN)^31^

## Results

The overall error rate from the literature meta-analysis (relying on data only from MRA-centric manuscripts) was 6.57% (95% CI: 5.51%, 7.72%). In comparison, the overall error rate for the ACT NOW CE Study was 1.04% (95% CI: 0.77%, 1.19%) based on the all-field calculation, which included all data elements regardless of case type (Table 1a). A difference of 5.53% (95%CI: 4.39%, 6.67%; p < 0.0001) was noted between the error rate estimates.

The ACT NOW CE Study error rate estimate for the populated-field meta-analysis was 2.57% (95% CI: 1.88%, 3.35%) (Table 1b). Similarly, this error rate was substantially lower than the 6.57% error rate from the literature (p < 0.0001).

## Discussion

Through the pooled analysis of data error rates from the literature, we were able to establish an average, overall MRA error rate – approximately 6.57% (or 657 errors per 10,000 fields). We compared this rate (resulting from the MRA meta-analysis) to the rates calculated for the ACT NOW CE Study (1.04% – 2.57% or 104 to 257 errors per 10,000 fields)^13^ and found that the error rates for the ACT NOW CE Study were substantially lower than those found in the peer reviewed literature – a difference of 553 (95% CI: 439, 667) per 10,000 fields (all-field total) and 400 (95% CI: 267, 533) per 10,000 fields (populated-field total). As cited in our previous work, we used both all-field and populated-field rates when calculating and presenting error rates for the ACT NOW CE Study, “to account for the variability in the calculation and reporting of error rates in the literature.”^13,36,37^ Based on these results, it appears that the MRA-QC framework implemented as part of the ACT NOW CE Study was successful in controlling MRA error rates.

Reports of clinical studies in the recent literature routinely lack descriptions of how the quality of the MRA was measured and controlled as well as the error rate ultimately obtained.^12^ For clinical studies that did report an error rate, substantial variability is noted in the way error rates were measured, calculated, and expressed.^12,37^ The ACT NOW CE Study was unique in that it implemented and evaluated formalized MRA training and continuous QC processes in an effort to improve data quality.^13,32^ To our knowledge, this was the first time that an MRA-QC framework, such as that published by Zozus and colleagues,^12,13,32^ was implemented and evaluated throughout the course of an ongoing clinical research study. There is a lack of evidence in the literature to suggest that previous clinical studies had implemented any formalized training or QC process to address error rates. As such, for this comparison, we made the decision (1) to limit to comparing against an overall error rate for each study rather than comparing rates across sites or over time; and (2) to provide both conservative (populated-field) and optimistic (all-field) measurements to account for variability across the literature. Given the variability and potential magnitude of the error rates from MRA, researchers should implement a formal data quality control framework that includes prospective quality assurance, such as abstraction guidelines^12^ with real-time error checks,^38^ abstraction training,^32^ and quality control during the abstraction process. These recommendations are echoed in the GCDMP chapter on *Form Completion Guidelines*.^39^

### Addressing Abstractor-related Variability

The reliance on human performance and associated underlying cognitive processes could be responsible for some or all of the variability and could be affected by the level of complexity of the data abstracted for a particular study. For example, the more cumbersome it is to identify, interpret, and collect a specific value from the EHR, the more likely for human error. The amount of abstractor-related variability in abstraction and quality control processes are likely residual effects of the traditional, bespoke and manual data management techniques that existed within the clinical research and clinical data management professions prior to the last two decades.^12,40,41^ Fundamentally, we recommend increasing standardization and QC of processes for capturing and processing data by qualified and trained research team members. The SCDM’s *Certified Clinical Data Management Exam*^™^ (CCDM) assesses a set of universal evidence-based, professional standards for individuals who manage data from clinical studies.^42^ Use of the CCDM exam as a tool for establishing competency could reduce variability universally. For example, the CCDM exam assesses (in those managing clinical data) the application of evidence-based practice and use of higher-order cognitive abilities (i.e., evaluation, synthesis, creation),^41,42^ potentially reducing variability in processes and human performance that were identified as potential sources of variability.

### Addressing Performance Improvement-related Variability

Empirical studies suggest that there is significant variability in the abstraction and quality control processes used;^43,44^ these different methods, process aids, and quality control activities could be responsible for the amount of variation observed in the error rates obtained from the literature. Several authors have further explored these underlying reasons for the high variability in abstraction.^43–49^ Further exploration on the causes of this variability is an important area for future research. In particular, the identification of human performance-related sources of variability with training-related root causes versus those caused by the abstraction tools and processes points to improvement interventions.^50–52^

### A Case for MRA Guidelines and Continuous QC

Although abstraction guidelines constitute a primary mechanism for preventing abstraction errors, they are not often used in clinical studies, which have traditionally relied on form completion guidelines. Until recent recommendations,^39^ clinical study form completion guidelines traditionally specified definition and format of fields and instructions for documenting exceptions, such as missing values, but usually stopped short of specifying locations in the patient chart from which to pull information, and acceptable alternative locations applicable across multiple clinical sites when the preferred source did not contain the needed data. The MRA-QC framework implemented as part of the data management activities for the ACT NOW CE Study addressed this limitation by developing standardized MRA training and abstraction guidelines with detailed instructions for locating each data point in the patient chart.^13,32^ Further, to account for the variability in clinical charting across institutions, secondary and tertiary locations within the EHR were also provided as alternatives, should the primary location not contain any relevant data. This approach offered clear and consistent instructions for all sites to follow, ensuring greater consistency and accuracy of the data collected.

The continuous QC process implemented as part of the MRA-QC framework, also offered an avenue for further clarification of the abstraction guidelines and periodic check-ins with each site to confirm consistency in interpretation of those guidelines. For example, the abstraction guidelines were updated significantly after the training.^32^ The guidelines were further updated following the routine quality control (independent re-abstraction) events where the root cause of errors was determined to be ambiguities in the abstraction guidelines.^13^

### A Case for Quantifying MRA Error Rates

It is unfortunate that the tendency to associate clinical research with rigorous and prospective data collection further fuels the perception that abstraction or chart review is not a factor in data accuracy when, in fact, (1) the chart itself and manual abstraction from the chart are the sources of most clinical research data error, and (2) manual abstraction from the chart (MRA) remains the most commonly used method for data collection.^12,26,47, 53–55^ Despite recommendations for measuring and monitoring MRA data quality,^44,48,56^ abstraction error usually remains unquantified in even the most rigorous clinical studies.^37,46,56,57^ Based on the now considerable evidence, we echo recommendations in the MRA Framework for abstractor training, tools, conducive environment and ongoing measurement and control of the MRA error rate^12^ and add to the calls for reporting data accuracy measures with research results.^58^ Reporting a data accuracy measure with research results should be expected in the same way that confidence intervals are expected; it is difficult, and not recommended, to interpret results in their absence.

### Limitations

Limitations specific to the analysis of error rates for the ACT NOW CE Study^13^ and the comprehensive literature review and meta-analysis^11^ have been presented separately and are not repeated here. Similar to the comprehensive literature review, we acknowledge limitations with the identification of MRA-centric manuscripts. As with any literature review, it is possible we may have missed relevant manuscripts due to a lack of standard terminology for data processing methods. Also, because our work is a secondary analysis, it relies on data that were collected for other purposes. Although we used error and field counts reported in the literature, prior work has shown that even these have significant variability.^33,36^

### Future Direction

While there is general agreement that the validity of research rests on a foundation of data, data collection and processing are sometimes perceived as a clerical part of clinical research. In between rote data entry and scientific validity, however, lie many unanswered questions about effective methods to ensure data quality, which, if answered, will help investigators and research teams balance cost, time, and quality while demonstrating that data are capable of supporting the conclusions drawn.

### Conclusion

Based on the comparison of the MRA error rate achieved under formalized quality assurance and process control to those reported in the literature, we conclude that such methods are associated with significantly lower error rates and that measurement and control of the data error rate is possible within a clinical study. We believe that the deployment of our MRA-QC framework allowed the ACT NOW CE Study to maintain error rates significantly lower than the overall MRA error rates identified in the relevant literature.

## Figures and Tables

**Table 1a.** Error Rate Comparison: MRA Literature vs. ACT NOW CE Study (All-Fields)

Groups	Error Rate (%) (95% CI)	I^2^
Medical Record Abstraction (MRA) Literature	6.57 (5.51, 7.72)	0.998
ACT NOW CE Study (All-Fields)	1.04 (0.77, 1.19)	0.889
Difference	5.53 (4.39, 6.67)	-

Note. CI = Confidence Interval; I^2^ = Higgins and Thompson’s I^2^ statistic for measuring the degree of heterogeneity, where ≤ 25%, indicating low heterogeneity; 25% – 75% indicating moderate heterogeneity; and > 75%, indicating considerable heterogeneity.^35^p-value < 0.0001

**Table 1b.** Error Rate Comparison: MRA Literature vs. ACT NOW CE Study (Populated-Fields)

Groups	Error Rate (%) (95% CI)	I^2^
Medical Record Abstraction (MRA) Literature	6.57 (5.51, 7.72)	0.998
ACT NOW CE Study (Populated-Fields)	2.57 (1.88, 3.35)	0.897
Difference	4.00 (2.67, 5.33)	-

Note. CI = Confidence Interval; I^2^ = Higgins and Thompson’s I^2^ statistic for measuring the degree of heterogeneity, where ≤ 25%, indicating low heterogeneity; 25% – 75% indicating moderate heterogeneity; and > 75%, indicating considerable heterogeneity.^35^p-value < 0.0001

## Data Availability

The datasets generated and/or analyzed during the current study are available in the NICHD Data and Specimen Hub (DASH) repository, https://dash.nichd.nih.gov/study/229026.

## Abbreviations

MRA: Medical Record Abstraction
QC: Quality Control
ACT NOW CE: Advancing Clinical Trials for Infants with Neonatal Opioid Withdrawal Syndrome Current Experience
NIH: National Institutes of Health
ECHO: Environmental influences on Child Health Outcomes
IDeA: Institutional Development Awards Program
ISPCTN: IDeA States Pediatric Clinical Trials Network
NICHD: National Institute of Child Health and Human Development
NRN: Neonatal Research Network
SCDM: Society for Clinical Data Management
GCDMP: Good Clinical Data Management Practices
CI: Confidence Interval
CCDM^™^: Society of Clinical Data Management’s Certified Clinical Data Management Exam^™^
EHR: Electronic Health Record
NAACCR: North American Association of Central Cancer Registries
HEDIS: Healthcare Effectiveness Data and Information Set
SDV: Source Data Verification
